# Ovarian Physiology and Pathology

**Published:** 1874-04

**Authors:** Alfred Meadows

**Affiliations:** F. R. C. P., London


*AMERICAN JOURNAL OF OBSTETRICS—November*, 1873:

Ovarian Physiology and Pathology.—*By Alfred Meadows, M.D., F. R. C. P., London.* — Having discussed these anatomical details, I endeavored to explain some points in the physiology of the ovaries—referring especially to the great changes which take place in their vascular system during ovulation. I also tried to explain by what means it appears to me that the phenomena of menstruation are brought about. I expressed my belief that the activity of the ovarian circulation induces a relaxed and dilated condition of the capillaries about the fundus and body of the uterus, in obedience to a well known law which governs functional and nutritional activity through the agency of the vaso-motor system; and I further expressed my conviction that the action of the ovaries upon the uterus in the production of menstruation is direct, and not reflex, and this opinion I grounded upon definite anatomical facts which admit of no dispute.

There is one observation which I would suggest, in passing, having reference to the analogy which exists between the diseases of the ovary and those of the testis. Some of the former are produced in precisely the same way, lead to the same results, are attended by very much the same symptoms, and require very similar treatment, as do the corresponding diseases of the testicles. Again, both organs are subject to various forms of inflammation, specific and non-specific, acute and chronic; and between orchitis, simple gonorrhœal, syphilitic, or due to any other form of blood-poisoning, there is indeed no essential difference, except it be in the greater intractability of the ovarian form, owing, as I believe, to the impossibility of securing physiological rest, and consequent freedom from vascular excitement, in the ovary—a condition which is at least possible of attainment in the case of the testicle. Again, in the simple neurotic affections, both are, I believe, subject to pretty much the same diseases. So, too, in regard to diseased conditions of the veins, it is now a well established fact that the pampiniform, or tubo-ovarian plexus of veins, the bulb of the ovary as it is also called, is liable to become varicose, and so to form a varicocele of the ovary precisely similar to that which occurs in the case of the testicle.

I think we ought to be able, by careful study of the various diseases to which the male organ is subject, to advance our knowledge of the obscurer forms of ovarian disease; and by the facility with which we can observe and watch the effects of remedies on diseases of the testis, whether applied externally or administered internally, if only we observe carefully, and are free from bias or prejudice, we ought by such practice greatly to advance ovarian therapeutics, and thus to remove what I cannot help feeling is an opprobrium to gynæcological science, viz., the present state of diagnosis and treatment of many of the commoner but obscure forms of ovarian disease.

In illustration of the pathological influence of the ovaries on distant parts, and the way in which that influence is exercised, it is difficult to make a selection from the many interesting cases which fall under one's observation; but I will select, for its novelty and importance, the *pathological influence of the ovaries upon the nervous system,* and I do so partly because the lessons which it teaches, at least if I correctly interpret them, have a much wider significance than appears at first sight, though I can only now suggest a line of thought which the experience of others may serve to illustrate.

Now, it would not be possible to exaggerate the interest and importance of this history. The question which presses for an answer is, how are we to explain the phenomena which occurred by reference to the assumed cause? In the first place, there were two factors in the case; there was the mental effect of fright on the one hand, and there was the disturbing influence of sudden acute menstrual suppression on the other. But, if the views which I have propounded here in regard to the physiology of menstruation, and the relative importance of the uterine and ovarian functions, have any foundation in fact, or are at all consistent with reason, then it must be admitted that we have hitherto attached far too much importance to the mere uterine part of menstruation—that is, to the discharge of blood and mucus from the uterine surface—and far too little to the influence which is borne by ovulation in the process. Yet, if it be true, as I have again and again insisted, that the ovaries are the very central point in the generative system, and that all the rest are mere appendages to them, it follows, I think, that, except for the purely *local* phenomena which may result from the disturbance of the proper uterine functions, no serious mischief can accrue to more distant parts from such disturbance. To my mind, the cessation of menstruation, regarding that as a mere sanguineous discharge from a particular organ, is not more likely to be attended by evil consequences to distant parts, than would be the arrest of any similar discharge from the bowel or elsewhere. It is different, however, with regard to the ovaries. We have seen how, physiologically, these organs are capable of influencing, not only particular parts at a distance, but even the entire organism, and how striking are the consequences of their removal. Physiology, as I have said, is twin-sister with pathology, and it is a law, I believe, in the animal economy, that the pathological importance of a part will bear a very strict comparison with its physiology in regard to the relative effects upon the system.

To the ovaries, then, we must look, at least in my judgment, for some explanation of the symptoms which occurred in the case I have cited, for they, in my opinion, were alone responsible. How, then, are the facts to be explained? It will be remembered that the amaurosis was at first limited to one eye, the right. Now, this fact alone, I think, is opposed to the view that the suppression of the menstrual discharge was the cause of the symptoms, for then surely not one but both eyes would have become affected, whereas, on the contrary, the result being thus, as it were, one-sided, the logical inference is that the cause which operated to produce the result was one-sided also. And this view is fully borne out by reference to other cases where serious nervous phenomena are brought about by causes acting at a distance from the nervous centres. I believe, in short, that this was a case of what is called *reflex paralysis,* the paralysis being limited to one optic nerve, and probably to that one opposite the side of the affected ovary. Now, that such a thing is possible I need not, I think, stay long to prove. I have had under my care at different times, several cases of complete amaurosis of both eyes, brought about entirely by the gradual arrest in the process of ovulation, first in one ovary, then in the other, until both had completely ceased to act, and this was shortly followed by absolute blindness which remained permanently and hopelessly incurable, owing apparently to the utter impossibility of restoring the ovarian function. I have exhausted all my stock of remedies in cases of this kind, and I know of no more distressing affections than these to treat.

I believe that, just as arrest of the secretion of bile and consequent jaundice may be produced by fright or other severe mental impression, so in like manner the function of ovulation may be suddenly arrested; and as there can, we believe, be no menstruation without ovulation, the arrest of the latter suspends the ovarian influence; the blood-vessels which supply the menstrual secretion by reason of their dilatation in the manner I have already explained, are suddenly contracted and the discharge ceases. At the same time, this sudden arrest of a most important function, (ovulation), just when it is at the crisis of its development, causes rapid contraction of the blood-vessels at some part of the spinal cord, the effect of which is transmitted to some distal extremity, and so a paralysis is produced. I know of no more interesting subject in ovarian pathology than this of the influence of the ovaries upon the nervous system, because it is obvious that if they are capable of thus affecting the nervous system, there is hardly any organ in the body which may not be brought under their influence.

I have already said that the ovary during the performance of its function of ovulation increases to at least double its size, that it becomes intensely vascular, is the seat of an actual hemorrhage into its substance, and finally has a distinct laceration of its tunics—and all this, probably, occurs again and again, not once now and then, but month by month, without giving rise to any disturbance, local or general; without, in fact, occasioning any symptoms whatever, except a discharge of blood from the uterus. Under these circumstances we have probably all the local phenomena usually attributed to a state of inflammation, with the single exception of *pain*—there is redness, swelling, and probably local elevation of temperature—yet no one will pretend to say that inflammation exists; at least, if it does, it is not of the kind which we ordinarily understand by the term *ovaritis*. Where, then, is the difference?

This is a difficult question to answer, and the more we think upon it the more, I believe, shall we be inclined to admit that with the single exception of *pain* and *tenderness* there is absolutely no sign or symptom. The very fact of the existence of pain implies something that is abnormal, for I may take it for granted that the performance of normal, healthy ovulation is a process which is perfectly free from pain. Upon what, then, does this pain depend? If we can determine this we shall probably have settled the question as to what is meant by the term *ovaritis*. Tenderness, we may take for granted, is a usual condition of the ovary during ovulation. Admitting the fact of tenderness as a usual result of ovulation, its occurrence is limited entirely to the time of ovulation; when that is over, all tenderness disappears; whereas, in the cases of so-called ovaritis, tenderness to touch remains as a constant quality, and pain, independently of touch, is superadded to it.

Is it, then, incorrect to assume that in the great majority of cases of ovarian inflammation, this morbid condition is the consequence of abnormal ovulation? No doubt you may get inflammation quite independently of ovulation as a cause—for instance, as in the testicle so in the ovary, inflammation may result directly from a gonorrhœa. Again, ovaritis may occur as a consequence of acute menstrual suppression from cold, and is a far more serious affair. Lastly, the most severe of all forms of ovaritis is that which occurs now and then in the course of parturition. I have met with instances of all these forms of the disease, and have, therefore, no doubt about their existence. But, while admitting all these varieties, I nevertheless believe that by far the larger number of cases of ovaritis are due, in the first instance, to some abnormal discharge of the ovarian functions, and I ground this opinion upon the following facts and arguments:

In the first place, it is consistent with analogy, and is borne out by reference to the pathological history of other organs, to believe, that perverted physiological action is a fertile source of disease.

Secondly, in the great majority of the cases of ovaritis, there is no history either of gonorrhœa, or of acute menstrual suppression, or of parturition, which can be referred to as a starting-point.

Again, ovaritis never occurs, at least I never met with or heard of a case, after the climacteric period or before puberty, that is to say, it seldom or never occurs before or after the establishment of the ovarian function of ovulation. This fact seems to me to imply that, inasmuch as the disease in question is met with only during menstrual life—in other words, during the performance of the function of ovulation—therefore, it is highly probable that in some way or other it is connected with functional activity. I have noticed further, that women who are married and have large families seldom or never suffer from ovaritis; but that, on the contrary, its frequency is in inverse proportion to the number of pregnancies, being much more frequent in those who have few or no children than in those who have many; and further, that it is most frequent of all in the unmarried between the ages of thirty and thirty-five, and in the married but sterile between the ages of twenty and thirty. What inference, then, can be drawn from these simple facts? The more common explanation of the last mentioned, which is found in books, is, that in the married of this class the sterility results from the ovaritis—and this no doubt is so far true; but then this only removes the question one point farther back, because we are still confronted with the inquiry: What is the cause of the ovaritis? Obviously, the cause of the inflammation *is the* TRUE *cause* of the sterility. The explanation which I venture to offer is, that it is caused by some flaw in the process of ovulation—a flaw which hinders the development of healthy ova, and which, at the same time, in some way leads to inflammation of the ovary; the non-development of healthy ova would, in itself, be a sufficient cause of sterility. It may be said, admitting the fact of the defective ovulation as a principal cause of ovaritis, the question remains as to what is the exact nature of this defect? Let me say at once that I am now in the land of speculation, and should wish, therefore, that what I say should be rigidly criticised, and, so far as is possible, tested by careful clinical observation and experience.

We are apt, I fear, in our gynæcological studies, especially in all that relates to menstruation, to pay too much attention to the uterus and too little to the ovary. I have already stated that in the process of ovulation the graafian follicle, with its contained ovum, gradually works its way from the more central portion of the ovary to some point on the surface whence it may escape; having arrived there in due course of its maturation, the outer covering bursts, the ovum escapes into the fallopian tube or oviduct, the blood-vessels, which up to this point have been enormously increased in size and number, are now relieved, and they speedily return to their wonted quietude until their services are again called into requisition.

Now, we do not know why, or rather we cannot explain exactly how, it comes to pass that ova should, as a rule, make their way to the free surface whence they may escape; but there is almost no rule without its exceptions, even in physiology, and we may well ask how it would fare with the ovary if, instead of going into the free surface, an ovum were to attempt to make its debut at the attached border. How are the vessels *then* to be relieved? What can the ovary effect to quiet the local excitement? One of two courses, we may suppose, will be taken: either the ovum will die, the follicle gradually shrink, and the vessels return to their normal state, or else the now morbid action will go on increasing, and may lead to disease in various forms, the very mildest of which will be a condition such as is described by the term *ovaritis*.

I have a patient under my care, who, having menstruated regularly and properly for two years, from nineteen to twenty-one, was seized with a severe attack of rheumatic fever, after which the catamenia ceased for five years entirely. Here it seems evident that the constitutional state interfered so seriously with the ovarian functions that they have never since been properly performed.

In another case, which has quite lately been under my care, * * * * as a child, she had been subject to strumous abscesses, and she had most unmistakable evidence of the strumous diathesis. * * * * *.

Now, the chief points to be noted in such a case as this are, first, the strongly marked diathetic condition; secondly, the existence of pain almost from the commencement of menstruation, and that too in the situation of one ovary; thirdly, the aggravation and subsequent irregularity, following exposure to cold; lastly—and the bearing of this is important, not only as confirming the accuracy of the original diagnosis, but also as indicating the general principle of therapeutics—the only remedies which proved of the slightest avail were such as aimed at the constitutional taint. From all which, I think we are justified in assuming that we had here to deal with a case of strumous inflammation of the ovary, or, I might say, a case of strumous ovulation.

